# Exploration of Cytotoxicity and Antibacterial Activities of M‐Ceo_2_ (M = Ag, Cu, Te, and Ta) Nanoparticles

**DOI:** 10.1002/open.202500278

**Published:** 2025-09-12

**Authors:** Faiza Qureshi, Suhailah S. Aljameel, Muhammad Nawaz, Mohammad Azam Ansari, Firdos Alam Khan, Sultan Akhtar, Mariam Ali Alsayed, Mohammad J. Akbar, Muzaheed

**Affiliations:** ^1^ Deanship of Scientific Research Imam Abdulrahman Bin Faisal University P.O. Box 1982 Dammam 31441 Saudi Arabia; ^2^ Department of Chemistry College of Science Imam Abdulrahman Bin Faisal University Dammam 31441 Saudi Arabia; ^3^ Department of Nano‐Medicine Research Institute for Research and Medical Consultations (IRMC) Imam Abdulrahman Bin Faisal University Dammam 31441 Saudi Arabia; ^4^ Department of Epidemic Diseases Research Institute for Research and Medical Consultations (IRMC) Imam Abdulrahman Bin Faisal University P.O. Box 1982 Dammam 31441 Saudi Arabia; ^5^ Department of Stem Cell Research Institute for Research and Medical Consultations (IRMC) Imam Abdulrahman Bin Faisal University P.O. Box 1982 Dammam 31441 Saudi Arabia; ^6^ Department of Biophysics Institute for Research and Medical Consultations (IRMC) Imam Abdulrahman Bin Faisal University Dammam 31441 Saudi Arabia; ^7^ Department of Pharmaceutics College of Pharmacy Imam Abdulrahman Bin Faisal University Dammam 31441 Saudi Arabia; ^8^ Department of Clinical Laboratory Science College of Applied Medical Sciences Imam Abdulrahman Bin Faisal University Dammam 31441 Saudi Arabia

**Keywords:** antibacterial, ceO_2_, cytotoxicity, nanoparticles

## Abstract

This study focuses on the ultrasonic synthesis of M‐CeO_2_ (M = Ag, Cu, Te & Ta) nanoparticles (NPs) and screening of their cytotoxicity and antibacterial activities. The prepared NPs are characterized by different techniques such as X‐ray diffraction, transmission electron microscope, SEM‐EDX,DR‐UV‐visible spectrophotometer, and dynamic light scattering analysis. The cytotoxicity of M‐CeO_2_ nanoparticles are assessed against cancer cells such as colorectal carcinoma (HCT‐116) and cervical cancer cells (HeLa) and non‐cancer cells (embryonic kidney cells HEK‐293). The effect of post‐48 h treatment of CeO_2_, Ag‐CeO_2,_ Cu‐CeO_2_, Te‐CeO_2_, and Ta‐CeO_2_, on HCT‐116 and HeLa cells showed a noteworthy reduction in cell viability. The treatments of Ag‐CeO_2_ also display a reduction in cancer cell viability but statistically not significant. The treatment of CeO_2_ shows better inhibitory action on HCT‐116 and HeLa cells. HEK‐293 is treated with CeO_2,_ Ag‐CeO_2,_ Cu‐CeO_2_, Te‐CeO_2,_ and Ta‐CeO_2_ NPs with the same dosages, there is a minor decline in the cell number, but the percentage of cells viability is greater than HCT‐116 and HeLa cells. The antibacterial activity of NPs against *E. coli* and *S. aureus* is tested, and Te‐CeO_2_ NPs show better antibacterial activity. The lowest MIC displayed by Te‐CeO_2_ is 0.25 mg mL^−1^ against *E. coli* and 4 mg mL^−1^ for *S. aureus*, respectively.

## Graphical abstract

Cu‐CeO_2_, Ag‐CeO_2_, Te‐CeO_2_, and Ta‐ CeO_2_nanoparticles (NPs) are synthesized by ultrasonic method and they demonstrate improved inhibitory action of_,_ on HCT‐116 and HeLa cells. The antibacterial activity of cerium oxide NPs against *E. coli* and *S. aureus* indicated Te‐CeO_2_ NPs being superior with MIC 0.25 mg mL^−1^ against *E. coli* and 4 mg mL^−1^ for *S. aureus*.

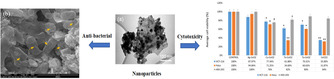

## 1 Introduction

Nanoparticles (NPs) are widely known for their beneficial attributes over their bulky counterparts and some nanomaterials have higher applicability than most NPs.^[^ ^1^ ^]^ While metal oxides NPs/nanosheets/quantom dots using Zn, Fe, Si, Al, Zr, Ti have been extensively and successfully used for many purposes.^[^ 2, 3, 4 ^]^ Their biological dexterity and the proven pharmaceutical efficacy against tumor has compelled researchers to unearth many such metal or metal oxide‐based potential leads as anticancer compounds like cadmium, indium, niobium, zinc, silver, gold, iron, silicon, Lanthanum, etc.^[^ 5, 6, 7, 8, 9, 10, 11, 12, 13, 14 ^]^

Nanoceria or cerium oxide nanoparticles (CONP), a recent innovative molecule that is taking over the metal oxide nanoparticle research in many fields but more notedly in anticancer therapy, is a dual‐capacity redox catalyst. Owing to its UV light‐absorbing abilities, use of CONP in surface coatings is very common.^[^ ^15^ ^]^ There are many pathways corroborated by research that shows nano CeO_2_ to be anti‐ or pro‐oxidation in regulating reactive oxygen species (ROS) levels in biological systems by acting as ROS‐related enzymes. The ROS‐related enzymes are responsible for cancer cell death by activating ROS production as response to change in physiological pH. The ability of CeO_2_ to mimic ROS‐related enzymes also helps with generation of molecular oxygen that improves the therapeutic regime, relieves tumor hypoxia, leading to tumor cell sensitization to improve photo‐ and radiation‐therapeutic results.

There are reports that confirm CONP are safe for drug delivery, tissue regeneration, gene therapy, theranostics, and medical imaging.^[^ ^16^ ^]^ Their cytoprotective abilities promote antioxidant capacity by countering cytotoxic nitric oxide donors and H_2_O_2_.^[^ ^17^ ^]^ Green/plant‐based synthesis significantly reduces toxicity to various organs and have no effect on antioxidative enzymes, total protein contents, lipid peroxidation, and nitrosative stress.^[^ ^18^ ^]^ Beside therapeutic efficacy, it has been reported to add to plant tolerance to environmental stress, such as salinity as observed in foliar application of CONP to Moldavian balm and spearmint plants.^[^ ^19^ ^,^ ^20^ ^]^

When used as a delivery carrier for a therapeutic agent, the combined cytotoxic outcome of the nanoceria and the drug has prominent result on anticancer therapy.^[^ ^21^ ^]^ The investigation into use of CONP as anticancer agent warrants sincere attention as there is too much that can work in favor of a novel CONP‐ based anticancer therapy. CONPs offer differential cytotoxicity by not affecting normal cells as well as offer protection against oxidative species through its antioxidant properties.^[^ ^22^ ^]^ CONPs are structured as cerium center inside oxygen lattice and their unique 3^+^/4^+^ state ratio has shown promising medicinal properties, depending on the different factors such as cell environment and pH.^[^ ^23^ ^,^ ^24^ ^]^ Be it the assorted abilities of CONP or their nominal toxicity to regular cells and tissues and/or their antioxidant potential, CONPs appear to be ideal candidate for treatment of cancer cells.

The anti‐invasive and protective properties of CONP also make nanoceria open to evaluate against other ROS‐ related diseases as well as against microbial infections. Many versions of CONPs have been researched with different synthesis methods and their antimicrobial effects.^[^ 25, 26, 27, 28 ^]^

Due to many exceptional characteristics, CONP demonstrates boundless potential in regenerative medicine and tissue engineering; CONP are known oxidoreductase‐ and phosphatase‐like nanozyme, and reportedly the first inorganic mitogen that stimulate regeneration by activating proliferation of cells and accelerate blastema growth. CONP reduces inflammation and autoimmune response, as well as possessing antimicrobial and anti‐biofilm properties, opening avenues for their use in cellular applications and biomedical technologies.^[^ ^16^ ^]^

The present study is directed at synthesis of metal‐doped CONPs and to investigate their cytotoxicity and antibacterial potential as the nano version of the cerium oxide outperforms its regular form by a huge margin. The selection of metals used here is based on their proven biological activities (Ag and Cu) and/or their non‐toxicity in biomedical applications and ability to boost charge transfer (Te and Ta).^[^ ^29^ ^]^ The *in‐vitro* analysis will compare the effect of these M‐CONPs with the pristine CONP on the cytotoxicity (against colon cancer and cervical cancer cell lines) and antibacterial efficiency. The study will add to the library of nanoceria therapeutics and will provide new method of synthesis and insight into its effect on potential anticancer and medicinal properties.

## 2 Results and Discussion

### 2.1 Characterization

**Figure 1:**
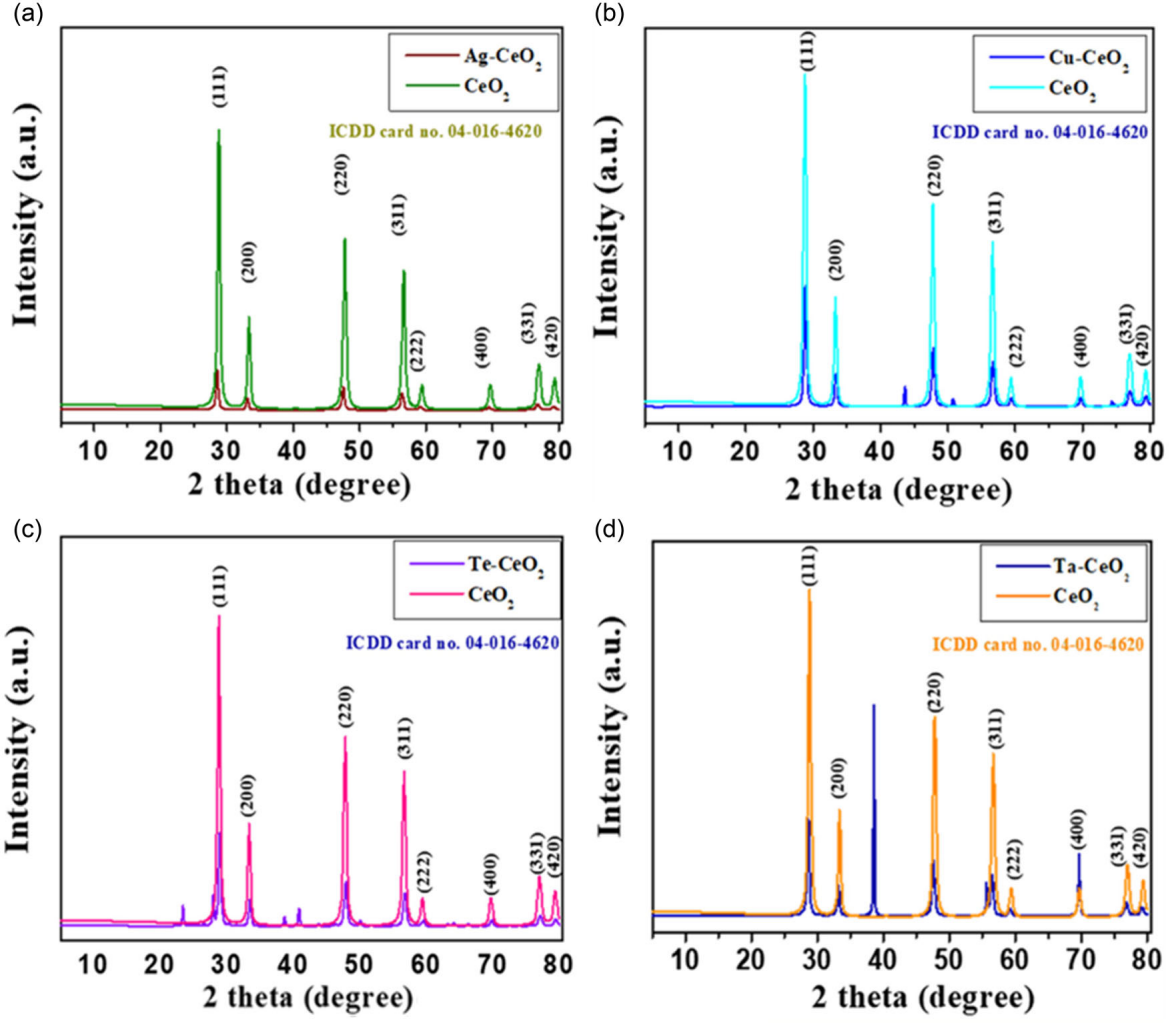
XRD patterns of a) Ag‐CeO_2_ NPs, b) Cu‐CeO_2_ NPs, c) Te‐CeO_2_ NPs, and d) Ta‐CeO_2_ NPs.

The XRD patterns of CeO_2_, Ag‐CeO_2_, Cu‐CeO_2_, Te‐CeO_2_, and Ta‐CeO_2_ NPs are displayed in the **Figure** **1**a–d. All peaks of CeO_2_, are indexed well with standard card (ICDD card no. 04‐016‐4620) confirming the formation of CeO_2_ nanoparticles with cubic phase and indicating successful preparation of CeO_2_ NPs. Peaks in Ag‐CeO_2_, Cu‐CeO_2_, Te‐CeO_2_, and Ta‐CeO_2_ nanoparticles matched well with CeO_2_, some extra peaks were observed due to the presence of Cu, Te or Ta in CeO_2_ NPs. Debye‐Scherrer (Equation 1) was employed to calculate the average crystalline sizes of CeO_2,_ Ag‐CeO_2_, Cu‐CeO_2_, Te‐CeO_2_, and Ta‐CeO_2_ nanoparticles and were found to be 19.22, 21.93, 21.91, 21.30, and 46.39 nm, respectively.

(1) D=0.9λβ/βcosθ

Here, *λ* represents the X‐ray wavelength, *β* denotes the full width at half maximum (FWHM), *θ* is the diffraction angle, and D indicates the particle diameter.

**Figure 2:**
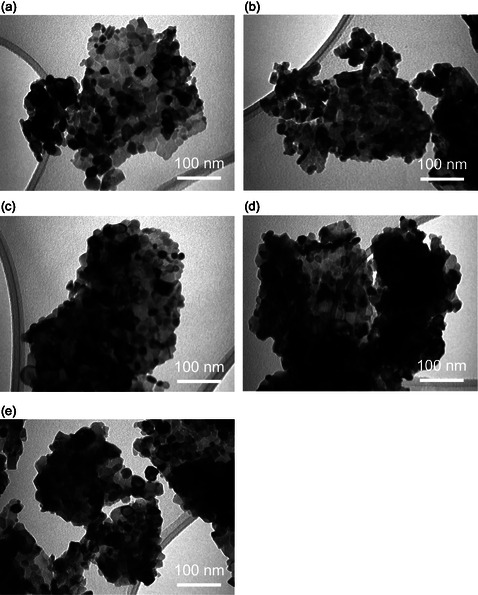
TEM images of a) individual CeO_2_ NPs, b) Ag‐CeO_2_ NPs, c) Cu‐CeO_2_ NPs, d) Te‐CeO_2_ NPs, and e) Ta‐CeO_2_ NPs. All scale bars are 100 nm.

**Figure 3:**
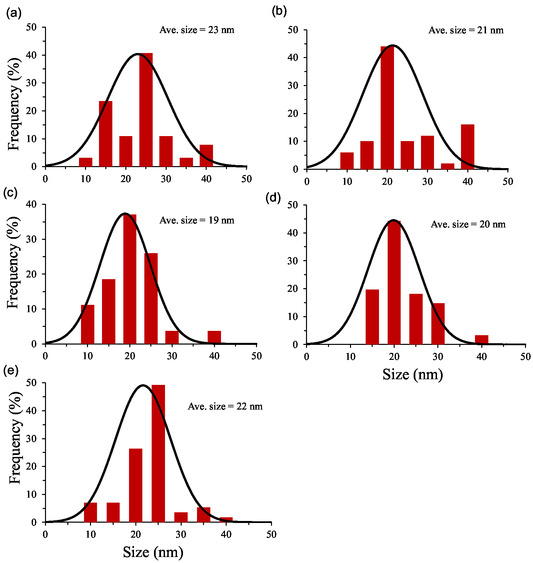
Size histogram along with average size (in nm) of a) individual CeO_2_ NPs, b) Ag‐CeO_2_ NPs, c) Cu‐CeO_2_ NPs, d) Te‐CeO_2_ NPs, and e) Ta‐CeO_2_ NPs.

Transmission electron microscope (TEM) analysis was performed to evaluate the surface morphology and structural features of the prepared nanoparticles. The representative images of each product are presented in **Figure** **2**a–e, and the sizes in the form of size histograms in **Figure** **3**a–e. As shown in the Figure 2, the surface morphology of individual CONP as well as the Ag, Cu, Te, and Ta incorporated CONP (Ag‐CeO_2_, Cu‐CeO_2_, Te‐CeO_2_, and Ta‐CeO_2_) is clearly visible. TEM images revealed that CONP displayed different shapes such as spherical, round, and hexagonal, with a moderate degree of size distribution. The morphology of the pure‐CeO_2_ specimen indicated that the nanoparticles were nearly monodispersed, stable, and maintained a well‐defined individual nature, suggesting good synthesis control. The average particle diameter was estimated to be around 20 nm, which falls within the expected nanometer scale for CeO_2_‐based systems (Figure 3).

Upon incorporation of Ag, Cu, Te, and Ta into CeO_2_, no significant alteration in the primary morphology of the nanoparticles was observed. However, a slight increase in aggregation or agglomeration tendency was noted in the doped systems. This behavior can be attributed to the introduction of doping ions (Ag, Cu, Te, and Ta), which may influence the surface energy and interparticle interactions, thereby promoting partial clustering. Such agglomeration is commonly observed in multi‐component nanoparticle systems due to changes in charge distribution and surface chemistry. Furthermore, the TEM analysis suggests that although the dopants did not drastically alter the size and shape of the CONP, they may influence crystallinity, surface defects, and interfacial properties, which are critical factors for their functional performance in catalytic or biomedical applications. The preservation of the nanoscale dimension along with slight modifications in aggregation indicates that the doping process was effective without compromising the fundamental morphology of CeO_2_.

**Figure 4:**
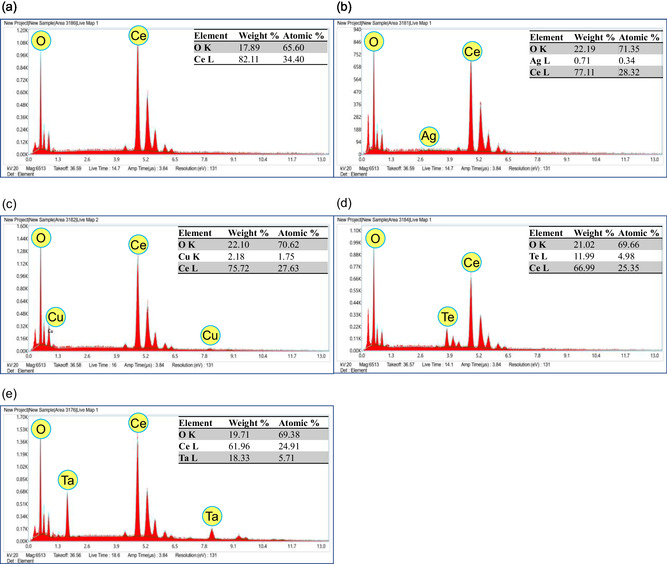
EDX spectra and elemental composition (in weight and atomic percentage) of a) individual ceO_2_ NPs, b) Ag‐CeO_2_ NPs, c) Cu‐CeO_2_ NPs, d) Te‐CeO_2_ NPs, and e) Ta‐CeO_2_ NPs.

**Figure 5:**
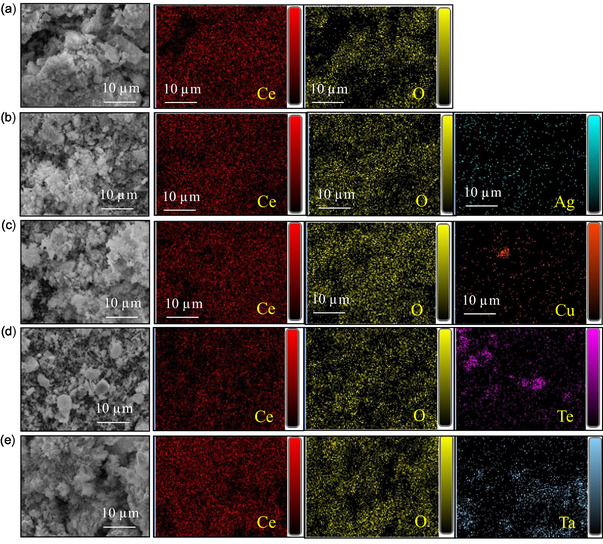
SEM and corresponding EDX mapping images of a) individual CeO_2_ NPs, b) Ag‐CeO_2_ NPs, c) Cu‐CeO_2_ NPs, d) Te‐CeO_2_ NPs, and e) Ta‐CeO_2_ NPs. All scale bars are 10 µm.

EDX analysis was carried out to verify the successful incorporation of Ag, Cu, Te, and Ta into the CONP. The EDX spectra, together with the corresponding elemental composition data and EDX mapping images, are presented in **Figure** **4** and **5**. For the pristine CONP, the spectrum showed distinct peaks corresponding to Ce and O, confirming their expected stoichiometric composition. In the case of doped specimens, additional elemental peaks were observed: the Ag‐CeO_2_ nanoparticles exhibited a characteristic Ag peak along with Ce and O, while Cu‐CeO_2_, Te‐CeO_2_, and Ta‐CeO_2_ samples displayed Cu, Te, and Ta peaks, respectively, confirming the presence of these dopants within the CeO_2_ matrix. The relative intensities of these peaks provide qualitative evidence of the successful incorporation of the respective elements.

Furthermore, the EDX mapping images reinforced these findings by illustrating the homogeneous distribution of Ag, Cu, Te, and Ta throughout the CeO_2_ nanoparticles, rather than isolated clusters or segregated phases. This uniform elemental dispersion suggests that the dopants were effectively integrated into the CeO_2_ structure during synthesis, which is a critical factor for achieving desired modifications in the physicochemical and functional properties. In summary, the EDX analysis confirmed the presence of the targeted dopant elements in the CONP, thereby reaffirming the success of the synthesis strategy. The incorporation of Ag, Cu, Te, and Ta without disrupting the fundamental CeO_2_ framework indicates the potential of these composite nanostructures for enhanced catalytic, optical, or biomedical applications, depending on the role of each dopant.

**Figure 6:**
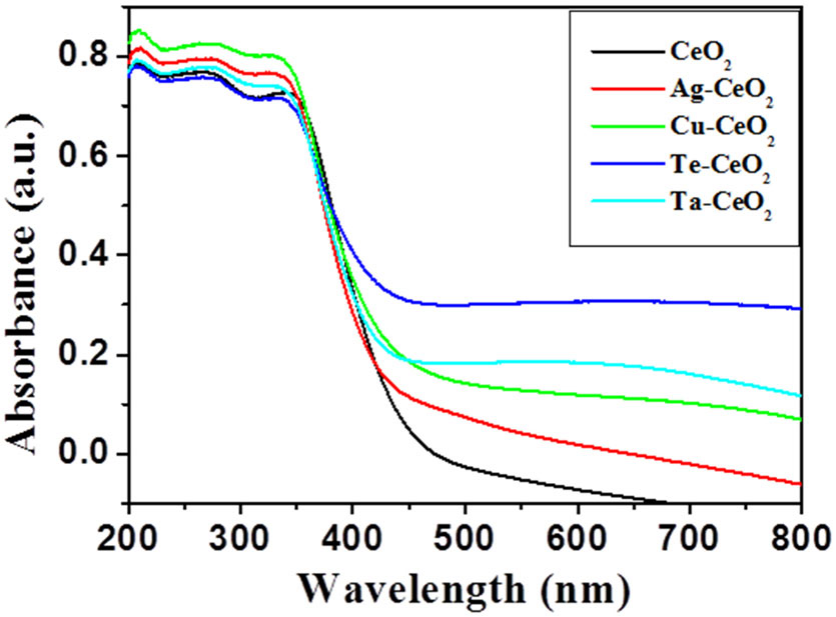
DR‐UV‐ spectra of CO_2_, ag‐CeO_2_, cu‐CeO_2_, te‐CeO_2_, and ta‐CeO_2_ nanoparticles.

**Figure** **6** shows the Diffuse Reflectance UV‐Visible spectroscopy (DR‐UV) spectra of CeO_2_, Ag‐CeO_2_, Cu‐CeO_2_, Te‐CeO_2_, and Ta‐CeO_2_ recorded in the range 200–800 nm. The nanoparticles exhibited spectra in the visible range, minor differences in the spectra were observed. The bandgaps of CeO_2_, Ag‐CeO_2_, Cu‐CeO_2_, Te‐CeO_2_, and Ta‐CeO_2_ nanoparticles were calculated using a plot of (*αhv)* ^2^ versu*s* photon energy (*hv)*, also called Tauc plot. The bandgap values of CeO_2_, Ag‐CeO_2_, Cu‐CeO_2_, Te‐CeO_2_, and Ta‐CeO_2_ nanoparticles were recorded 3.03, 3.14, 3.10, 3.0, and 3.02 eV, respectively.

The stability of the CeO_2_, Ag‐CeO_2_, Cu‐CeO_2_, Te‐CeO_2_, and Ta‐CeO_2_ nanoparticles was assessed from the zeta potentials, indicating the surface charge on the NPs. Zeta potential of CeO_2_, Ag‐CeO_2_, Cu‐CeO_2_, Te‐CeO_2_and Ta‐CeO_2_ nanoparticles were noted 5.62 ± 6.84, 20.2 ± 4.51, 17.6 ± 5.24, −1.29 ± 3.22, −2.16 ± 4.76 mV respectively (**Table** **1**). The results demonstrated that Ag‐CeO_2_ nanoparticles had a high dispersion stability, pursued by Cu‐CeO_2_ nanoparticles. High zeta potential implies increased surface change and thus stronger electrostatic repulsion, preventing the colloidal NPs encountering each other and reducing the agglomeration. The average particle size of CeO_2_ nanoparticles was recorded 883 nm with PDI 0.610. Whereas in case of Ag‐CeO_2_, Cu‐CeO_2_, Te‐CeO_2_, and Ta‐CeO_2_ nanoparticles, the average particle size was 333 nm (PDI: 0.364), 344 nm (PDI: 0.429), 347 nm (PDI: 0.544), 439 nm (PDI: 0.426), respectively. The variation between the particle sizes observed in TEM and dynamic light scattering (DLS) could be recognized because TEM provides images of the individual particles while DLS assesses the nanoparticle hydrodynamic radius. Furthermore, DLS implies the analysis of nanoparticle in a liquid solution and is therefore affected by aggregation or accumulation.^[^ ^30^ ^,^ ^31^ ^]^ The polydispersity index (PDI) was also determined to find that nanoparticle particle size distribution is polydisperse or monodisperse. As described by Honary et al., particles are classified as polydisperse or monodisperse based on their polydispersity index value whether it is above or below 0.7.^[^ ^32^ ^]^ As evident from Table 1, the PDI value of CeO_2_, Ag‐CeO_2_, Cu‐CeO_2_, Te‐CeO_2_, and Ta‐CeO_2_ nanoparticles was noted 0.610, 0.364, 0.429, 0.544, and 0.426, respectively indicating the monodispersity of the nanoparticles.

**Table 1:** Zeta potential, particle size, and polydispersity index of synthesized nanoparticles.

Nanoparticles	Zeta potential [mV]	Average particle size [nm]	Polydispersity index [PDI]
CeO_2_	5.62 ± 6.84	883	0.610
Ag‐ CeO_2_	20.2 ± 4.51	333	0.364
Cu‐ CeO_2_	17.6 ± 5.24	344	0.429
Te‐ CeO_2_	−1.29 ± 3.22	347	0.544
Ta‐ CeO_2_	−2.16 ± 4.76	439	0.426

### 2.2 Cytotoxicity Study

**Figure 7:**
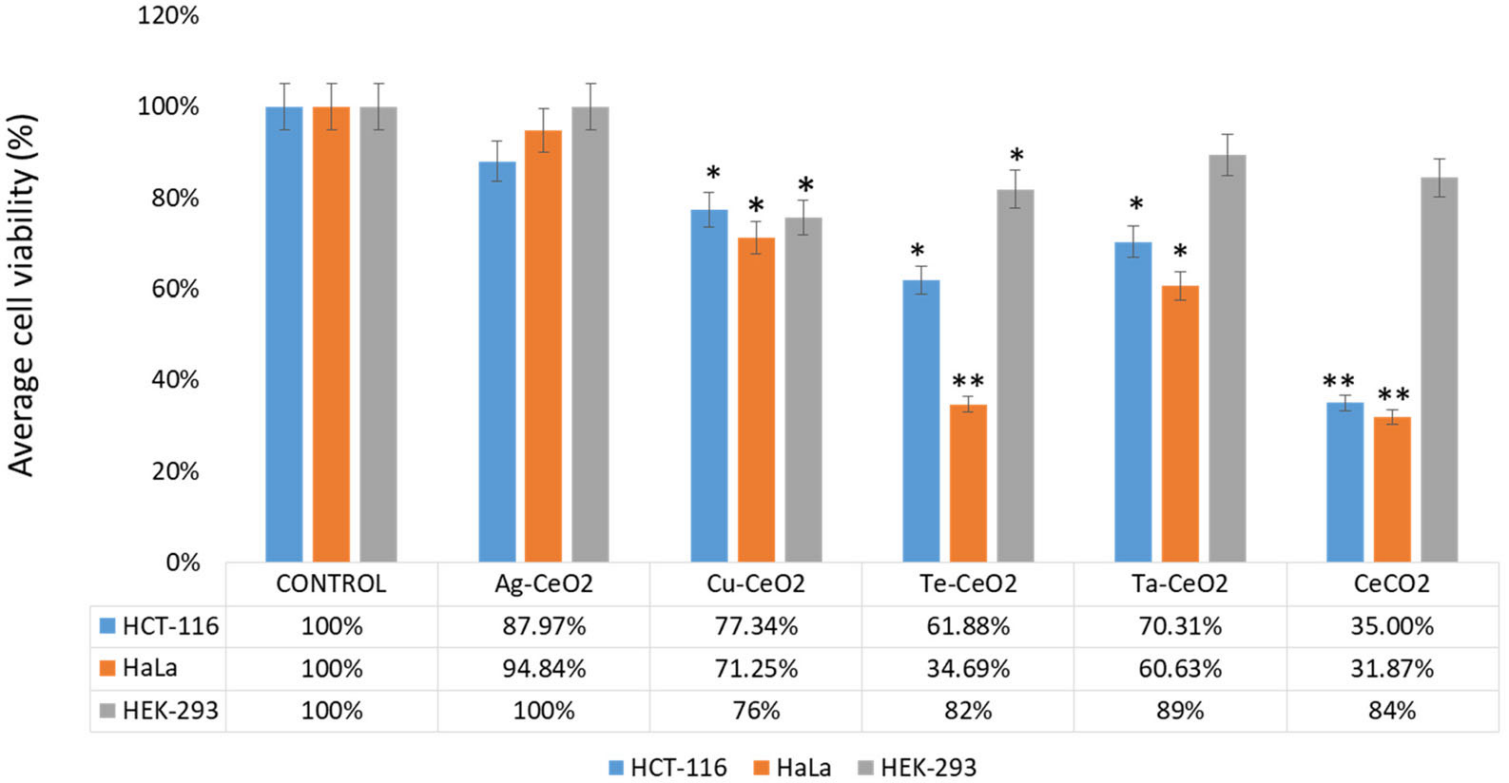
Effect of Ag‐CeO_2,_ Cu‐CeO_2_, Te‐CeO_2,_ Ta‐CeO_2,_ and CeO_2_ on cancer cells (HCT‐116, HeLa) and normal cells (HEK‐293) cells examined by MTT assay. *p < 0.005; **p < 0.001.

The effect of post‐48 h treatment of Ag‐CeO_2,_ Cu‐CeO_2_, Te‐CeO_2,_ Ta‐ CeO_2_, and CeO_2_ on HCT‐116 and HeLa cells showed a noteworthy reduction in cell viability. The treatments Ag‐CeO_2_ also proven a decline in cancer cell viability but statistically not significant (**Figure** **7**). The treatment of CeO_2_ demonstrated superior inhibitory action with compared to Cu‐CeO_2_, Te‐CeO_2_, Ta‐ CeO_2,_ on HCT‐116 and HeLa cells (Figure 7).

HEK‐293 were treated with Ag‐CeO_2_, Cu‐CeO_2_, Te‐CeO_2_, Ta‐CeO_2_, and CeO_2_ with the same dosages, there was a minor decline in the cell number, but the percentage of cells viability was greater than HCT‐116 and HeLa cells (Figure 7). This advocates that Ag‐CeO_2_, Cu‐CeO_2_, Te‐CeO_2_, Ta‐ CeO_2_, and CeO_2_ were more toxic to cancerous cells than HEK‐293cells. In this study, we have reported the anti‐cancer impact of Ag‐CeO_2__,_ Cu‐CeO_2_, Te‐CeO_2_, Ta‐ CeO_2_, CeO_2_ on HCT‐116 and HeLa cells.

The effect of post‐48 h treatment of CeO_2_ nanoparticles on HCT‐116 and HeLa cells showed a noteworthy reduction in cell viability. The cytotoxic effect of CeO_2_ nanoparticles may be due to programmed cell death or apoptosis. There are several studies which report that treatment of CeO_2_ nanoparticles induced cytotoxicity on HepG2, MCF‐7 cells, colorectal cancer cell and MG‐63 malignant cells.^[^ 33, 34, 35, 36 ^]^ The cytotoxicity of CeO_2_ may be due to antioxidant caused by redox switching mechanism and apoptosis.^[^ ^37^ ^]^ The treatment Ag‐CeO_2_ nanoparticles on HCT‐116 and HeLa cells showed a better cytotoxic action compare to treatment of CeO_2_ alone, which suggest that conjugation of Ag increased the cytotoxic effect on the cancer cells. The possible explanation of impact of Ag is due considerably increase in the frequency of apoptotic cells, increased cell cycle arrest at the S phase noteworthy reduction in cell viability and activating cell death cascade as reported in number of studies.^[^ 38, 39, 40 ^]^ Furthermore, the mechanism of action of Ag nanoparticles in cancer treatment has been reported to be associated with their ability to induce cell death through several pathways including the generation of ROS molecules that can cause DNA damage, which eventually lead to cell apoptosis in HCT‐116 and HeLa cancer cells.^[^ ^41^ ^]^ Furthermore, it was found that Ag nanoparticles can induce autophagy of cancer cells through activation of the phosphatidylinositol 3‐kinase pathway.^[^ ^42^ ^]^ In addition, it was reported that cancer cells have enhanced permeation and higher accumulation of Ag nanoparticles in cancer cells.^[^ ^43^ ^]^

### 2.3 Cancer DNA Disintegration

**Figure 8:**
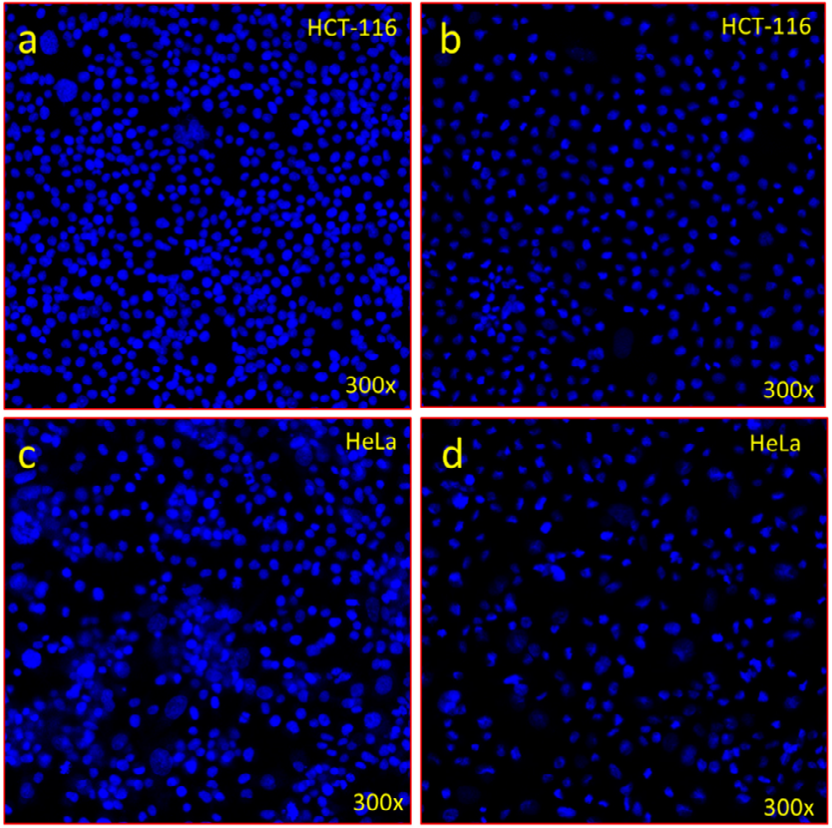
DAPI staining of HCT‐116 and Hela cells shows the influence of ceO_2_ on cancer cells. a) (control group without treatment) shows healthy cells. b) CeO_2_‐treated cells. c) The control cells of Hela shows healthy cells, d) shows that CeO_2_‐treated cells showed apoptotic cell death. Magnifications 300x.

The effect of CeO_2_ on cancer cells showed a substantial decline in HCT‐116 cells as shown by DAPI‐staining (**Figure** **8**b) as compared to control cells (Figure 8a). The treatment of CeO_2_ also caused a noteworthy decline in HeLa cells as shown by DAPI‐staining (Figure 8d) with compared to control group cells (Figure 8c). The decline in the cancer cells post‐treatment is owing to apoptosis.^[^ ^1^ ^,^ ^44^ ^]^

### 2.4 Antibacterial Activity

The antimicrobial activity of CONP has been reported; however, there are no reports on the antibacterial activity of Te‐CeO_2_ NPs. The antibacterial activity of NPs against *E. coli* and *S. aureus* pathogens was found out by standard broth dilution methods, results are presented in **Table** **2**. It was found that Te‐CeO_2_ NPs showed better antibacterial activity amongst tested NPs. The lowest MIC was shown by Te‐CeO_2_, which was 0.25 mg mL^−1^ against *E. coli* and 4 mg mL^−1^ for *S. aureus*. The MIC of positive control ampicillin were 2 and 1 µg mL^−1^, respectively against *E. coli* and *S. aureus*. Our findings on antibacterial activity of NPs aligns well with previous reports, which indicated that commercial and biosynthesized Te(0) NPs inhibit growth of *E. coli* at a concentration of 7 mg mL^−1^, but no effect was reported against *S. aureus* at the same dosage as measured by zone of inhibition assay.^[^ ^45^ ^]^ The Te‐CeO_2_ NPs in this investigation shown had a greater antibacterial impact against Gram‐negative *E. coli* than against Gram‐positive *S. aureus* bacteria and this is again consistent with the findings of Ao et al. Overall antibacterial activity is also significantly influenced by a variety of physiological and chemical external factors. The antibacterial action of CONP depends on their characteristics such as particle size, shape, pH, medium and synthesis methods, and particular bacterial strains involved. Typically, large surface areas, strongly reactive facets, and relatively high concentrations confer substantial toxic effects to CONP.^[^ ^46^ ^]^ Kuang et al. found that nano‐scale CeO_2_ has higher effectiveness against bacteria compared to bulk CeO_2_.^[^ ^47^ ^]^ Pelletier et al. examined the effects of a wide range of CONP parameters, such as size of particles, concentration, pH, type of bacterial strains and medium, and they found that both Gram‐positive and Gram‐negative bacterial strains were inhibited in a size‐ and concentration‐dependent manner.^[^ ^48^ ^]^

**Table 2:** MIC value (mg mL^−1^) of NPs against *E. coli* and *S. aureus*.

Nanoparticles	*E. coli*	*S. aureus*
CeO_2_	4	16
Ag‐CeO_2_	8	8
Cu‐CeO_2_	8	8
Te‐CeO_2_	0.25	4
Ta‐ CeO_2_	16	16

### 2.5 Morphological and Structural Analyses of *E. coli* Cells (SEM and TEM Analyses)

**Figure 9:**
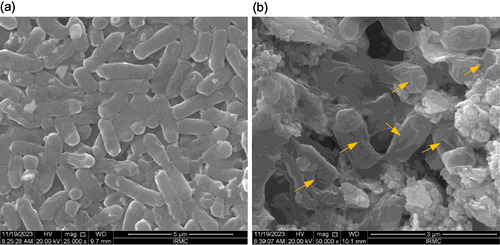
SEM micrographs displaying morphological changes in *E. coli* cells a) in the absence (control) and b) in the presence of Te‐CeO_2_ NPs.

The antibacterial results were used as a basis to further study the effects of Te‐CeO_2_ on the morphology of *E. coli* using SEM. **Figure** **9**a shows that the *E. coli* cells that were not treated maintained their original, characteristic, and elongated shape, with a smooth cytoplasmic membrane. However, *E. coli* cells that were exposed to Te‐CeO_2_ NPs revealed serious damage, with the cell membrane and wall appearing fragmented, deformed, inconsistent, and bumpy. This indicates an impairment of cellular membrane integrity, ultimately resulting in cell death (Figure 9b). In a previous study, it has been reported that CONP have been shown to cause substantial damage to the *E. coli* cell wall and membrane.^[^ ^49^ ^]^

**Figure 10:**
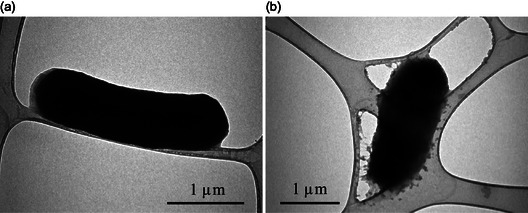
TEM images of the *E. coli* cell. a) The membrane of the control cells was intact and they were normal. b) The Te‐CeO_2_ NPs‐treated cells were aberrant and lost their integrity due to damage to the cell membrane, which indicates NPs internalization.

Additionally, the impact of NPs on the ultrastructure of *E. coli* cells was investigated using TEM examination (Figure 8). The untreated *E. coli* cell exhibited typical cellular characteristics, such as a rod‐shaped morphology, regularity, smoothness, and an intact cell wall and membrane (**Figure** **10**a). However, significant changes in the microscopic structure, such as severe damage to the cell wall and membrane, were seen in *E. coli* cells exposed to NPs. Furthermore, it was noted that the treated cells exhibited abnormalities, irregularities, and significant damage. In addition to cellular wall destruction, there was also observed a clear separation and disintegration of the cell wall and membrane from the cell (Figure 10b). TEM analysis reveals that the NPs are adsorbed, internalized, and penetrate the cells, resulting in the rupture of the cell membrane. This leads to the leakage of cytoplasmic content and collapse of the cell wall and membrane integrity, ultimately resulting in cell death (Figure 8b). Though, the exact mode of action of Te‐CeO_2_ NPs is still not clear. It has been reported that once bacteria are exposed to NPs, the NPs’ antibacterial action is triggered by their direct interaction with the bacterial membranes. Positively charged CONPs have been proposed to be easily adsorbed onto bacterial membranes by electrostatic attraction, which disrupts the membrane.^[^ ^46^ ^]^ Further, it has been demonstrated that the primary cause of CONP toxicity is oxidative stress brought on by the production of ROS. Because the ROS chemically break down a variety of macromolecules in bacteria, including as DNA, RNA, and proteins, thus they may severely damage bacterial cells.^[^ ^50^ ^,^ ^51^ ^]^

## 3 Conclusion

M‐CeO_2_ (M = Ag, Cu, Te & Ta) nanoparticles were synthesized successfully by ultrasonic method and characterized by XRD, TEM, scanning electron microscopy with energy dispersive X‐Ray analysis (SEM‐EDX), , (DR‐UV‐Visible spectrophotometer) and DLS analysis. The treatment of CONP demonstrated improved inhibitory action of Cu‐CeO_2_, Ag‐CeO_2_, Te‐CeO_2_, Ta‐ CeO_2_, on HCT‐116 and HeLa cells. The prepared nanoparticles show less cytotoxicity toward non‐cancer cells, HEK‐293. This advocates that Ag‐CeO_2_, Cu‐CeO_2_, Te‐CeO_2,_ Ta‐CeO_2_, and CeO_2_ were more toxic to cancerous cells than HEK‐293 cells. The antibacterial activity of NPs against *E. coli* and *S. aureus* pathogens indicated that Te‐CeO_2_ NPs has superior antibacterial activity amongst tested CONPs with MIC 0.25 mg mL^−1^ against *E. coli* and 4 mg mL^−1^ for *S. aureus*. The exceptional properties of CONP, as demonstrated by the literature and this research work warrants further investigations and unlocking the complete potential of CONPs in many fields, but relevant to this research, in cellular and tissue level biomedical technologies.

## 4 Experimental Section

4.1

4.1.1

##### Ultrasonic Synthesis of CeO_2_, Ag‐CeO_2_, Cu‐CeO_2_, Te‐CeO_2_, and Ta‐CeO_2_ Nanoparticles

1.0 g of cerium nitrate was dissolved in 30 mL of deionized water, afterwards 0.4 g trisodium citrate and 0.8 g urea was added to the cerium nitrate solution. After stirring, reaction mixture was probe sonicated for 40 min. After centrifuging, washing with water and methanol, the product was dried in an oven overnight.

To prepare the Ag‐CeO_2_, Cu‐CeO_2_, Te‐CeO_2_, and Ta‐CeO_2_ nanoparticles, 0.5 g CeO_2_ was dispersed in 20 mL of deionized water, then 0.25 g silver or copper or tellurium or tantalum power was added. After stirring at room temperature, reaction mixture was probe sonicated for 40 min. The product was centrifuged, washed, and dried to give Ag‐CeO_2_, Cu‐CeO_2_, Te‐CeO_2_, and Ta‐CeO_2_ nanoparticles.

##### Characterization

X‐ray diffraction (XRD, Rigaku) was employed to the crystal structure and phases of the as prepared nanoparticles. The structure and morphology of the prepared nanoparticles (pure CeO_2_, were evaluated by TEM (FEI TEM, Morgagni 268, Czech Republic), and (SEM‐EDX) (TESCAN, Vega 3 SEM, Czech Republic) was used to verify the presence of different elements and the chemical composition of the prepared nanoparticles. The TEM samples were prepared by depositing a drop of powder dispersion onto TEM grids and thereafter the grids were introduced into TEM to record images. For SEM‐Diffuse Reflectance EDX, a few drops from each sample were mounted onto metallic stubs having double‐sided carbon‐tape. TEM was operated at 80 kV and SEM images were taken at 20 kV. The UV‐visible spectrophotometer (JASCO‐V‐750) was operated to attain the diffuse reflectance spectrum (DRS) of the nanoparticles. DLS was performed on a Nano ZS Malvern Zetasizer instrument.

##### Cytotoxicity Study

Two cancer cells such as colorectal carcinoma HCT‐116 and cervical cancer cells HeLa were purchased from ATCC, USA were used to examine the anti‐cancer activity of Ag‐CeO_2,_ Cu‐CeO_2_, Te‐CeO_2,_ Ta‐ CeO_2,_ CeO_2_. We also took embryonic kidney cells HEK‐293 was used as a non‐cancer cells. For MTT assay, the cells were cultured in the media: DMEM/FBS, penicillin, and streptomycin in the CO_2_ incubator at a temperature of 370C. Cells with 6 × 104 cells/ml concentration were seeded in 96‐well cell culture plates and incubated again in the CO_2_ environment. The cells were treated (2.0 µg to 20 µgmL^−1^) with Ag‐CeO_2_, Cu‐CeO_2_, Te‐CeO_2_, Ta‐ CeO_2_, and CeO_2_ for 48 h. In the control group, Ag‐CeO_2_, Cu‐CeO_2_, Te‐CeO_2_, Ta‐ CeO_2_, and CeO_2_ for 48 h. Notably, NPs were not added to the control group. Now, 20 μl of MTT was added to each well and incubated for 4 h. The media was then changed with dimethyl sulfoxide and each well was measured by ELISA plate reader at working wavelength of 570 nm. The cell viability in percentage (CV%) was estimated using the following simple relation.^[^ ^1^ ^]^

(2) CV(%)=(Dt/Dc)×100

where (*Dt*) is the optical density of NP‐treated cells and (*Dc*) density of control cells (cells without any treatments), respectively.

In brief, cells were cultured and were taken for MTT assay.^[^ ^1^ ^]^ The cells were treated (2.0 µg to 20 µg mL^−1^) with Ag‐CeO_2_, Cu‐CeO_2_, Te‐CeO_2_, Ta‐ CeO_2_, CeO_2_ for 48 h. In the control group, Ag‐CeO_2_, Cu‐CeO_2_, Te‐CeO_2_, Ta‐ CeO_2_, CeO_2_ was not added. Then were added with MTT for 4 h and were read using a Plate Reader at 570 nm wavelength.^[^ ^1^ ^]^

##### DAPI Nuclear Staining

We have selected the sample CeO_2_ that displayed the maximum inhibitory action on cancer cells. HCT‐116 and HeLa cells were used to study the morphology of cancer cell nuclei. DAPI is a nuclear stain that specifically binds with nuclear DNA and is used as the marker for apoptosis. The cancer cells were treated with CeO_2_ with a dose of 20 µg mL^−1^ for 48 h and examined by DAPI staining assay and then visualized by using a confocal scanning microscope Zeiss, Germany.^[^ ^1^ ^]^

##### Antibacterial Activity

*E. coli* (ATCC 25922) and S. aureus (ATCC 25923) were procured commercially from ATCC, USA. The MIC microbroth dilution method was used to compute the MIC of the NPs as described by Qureshi et al.^[^ ^1^ ^]^ The bacterial cultures were incubated at 37 °C for 24 h after being exposed to twofold serial dilutions of NPs (*i.e*., 32−0.125 mg mL^−1^). The initial concentration of NPs at which no perceivable growth was observed is called the MIC value. Ampicillin (50 μg mL^−1^) served as a positive control.

##### SEM Morphology of *E. coli* Treated with MCT NPs

The topological alterations in *E. coli* following treatment with 0.125 mg mL^−1^ of NPs were examined by SEM. Following treatment and incubation, the samples underwent a 15 min centrifugation, and the pellets that were recovered were subsequently washed with PBS. After fixation with glutaraldehyde (2.5%) and osmium tetroxide (1%), the samples were dehydrated using a series of ethanol concentrations: 20, 30, 40, 50, 60, 70, 80, and 90%. This process was repeated once for each concentration and twice at 100% for 10 min each. The samples were then placed on the aluminum stubs, and finally, a gold coating was applied. Lastly, a SEM operating at 20 kV was used to examine how NPs affected the structure of the bacteria.^[^ ^52^ ^]^

##### Ultrastructural Alteration in *E. coli* Cells Caused by NPs: TEM Analysis

TEM was used to further examine the ultrastructural changes that occurred in *E. coli* following NP treatment. Centrifugation was used to pellet freshly produced colonies. After being treated with NPs, the cells (10^6^ CFU mL^−1^) were cultured for 16 h at 37 °C. The cells that were not treated with NPs were used as the control group. Subsequent incubation, pellets were collected, fixed (glutaraldehyde, 2.5% and osmium tetroxide, 1%), and dehydrated.^[^ ^52^ ^]^ Both treated and untreated samples were rinsed twice. The samples were then immersed in resin for an overnight period to allow for polymerization. Next, a microtome diamond knife was used to cut extremely thin slices, which were subsequently mounted carbon‐coated copper grids being stained with uranyl acetate and counterstained with lead citrate (4%). Lastly, TEM, was used to record the ultrastructural modification caused by NPs in *E. coli* cells.

##### Statistical Analysis

The results were obtained from triplicates and statistically assessed by Graph‐Pad Prism Software, USA.

## Conflict of Interest

The authors declare no conflicts of interest.

## Author Contributions

**Faiza Qureshi**: conceptualization; methodology; resources; writing—original draft preparation; writing—review and editing; supervision; project administration; funding acquisition. **Suhailah S. Aljameel**: writing—original draft preparation. **Muhammad Nawaz**: conceptualization; methodology; validation; investigation; resources; data curation; writing—original draft preparation; writing—review and editing; supervision; project administration; funding acquisition. **Mohammad Azam Ansari**: validation; formal analysis; investigation; data curation; writing—original draft preparation. **Firdos Alam Khan**: validation; formal analysis; data curation; writing—original draft preparation. **Sultan Akhtar**: formal analysis; data curation. **Mariam Ali Alsayed**: formal analysis. **Mohammad J. Akbar**: writing—review and editing. **Muzaheed**: writing—review and editing. All authors have read and agreed to the published version of the manuscript.

## Data Availability

The data that support the findings of this study are available from the corresponding author upon reasonable request.
